# The Effect of Powder Re-Use on the Coalescence Behaviour and Isothermal Crystallisation Kinetics of Polyamide 12 within Powder Bed Fusion

**DOI:** 10.3390/polym16050612

**Published:** 2024-02-23

**Authors:** Benjamin Sanders, Edward Cant, Catherine A. Kelly, Michael Jenkins

**Affiliations:** 1 School of Metallurgy and Materials, University of Birmingham, Elms Road, Birmingham B15 2SE, UK; bls715@student.bham.ac.uk; 2 The Manufacturing Technology Centre, Ansty Park, Coventry CV7 9JU, UK; edward.cant@the-mtc.org; 3 Independent Researcher, Worcester, UK; catherine.a.kelly3@gmail.com

**Keywords:** powder bed fusion, polyamide-12, Avrami, polycondensation, cross-linking, powder re-use

## Abstract

Polymer powder bed fusion (PBF) is becoming increasingly popular for the fabrication of lightweight, high-performance parts, particularly for medical and aerospace applications. This study investigates the effect of powder re-use and material aging on the coalescence behaviour, melt flowability, and isothermal crystallisation kinetics of polyamide-12 (PA-12) powder. With increased powder re-use, a progressive reduction in melt flowability and material coalescence is observed; at 200 °C, the particle consolidation time increases from 15 s in virgin powder to 180 s in powder recovered from build 6. The observed changes in the behaviour of PA-12 were attributed to polycondensation and cross-linking; these aging phenomena also create structural defects, which hinder the rate and extent of primary crystallisation. At an isothermal crystallisation temperature of 165 °C, the crystallisation half-time increased from 12.78 min in virgin powder to 23.95 min in powder re-used across six build cycles. As a result, the commonly used Avrami model was found to be unsuitable for modelling the crystallisation behaviour of aged PA-12 powder, with the co-efficient of determination (R^2^) reducing from >0.995 for virgin powder to as low as 0.795 for re-used powder. On the other hand, an alternative method, the Hay model, is able to successfully track full phase transformation within re-used powder (R^2^ > 0.99). These results highlight the importance of selecting the most appropriate model for analysing the crystallisation kinetics of PA-12 powder re-used across multiple build cycles. This understanding is crucial for obtaining the strong mechanical properties and dimensional precision required for the fabrication of functional, end-use parts within PBF.

## 1. Introduction

Laser sintering (LS) is a subset of additive manufacturing (AM), whereby parts are fabricated layer by layer, from a powdered polymeric material, using a three-dimensional computer-aided design (3D-CAD) geometry file [1,2,3]. LS of semi-crystalline, thermoplastic materials, most commonly polyamide-12 (PA-12) [2,4,5,6,7,8,9], has become increasingly popular for low-volume production of end-use parts. Application areas include medical (e.g., customised prothesis), aerospace (e.g., complex impellers), and retail (e.g., innovative insoles for trainers).

LS can offer complex, customised parts with high dimensional precision. Another key advantage is lightweighting and the fabrication of parts with a high strength-to-weight ratio [1,3,10,11,12]. Given the climate crisis, it is becoming increasingly necessary for the manufacturing industry to transition toward more sustainable processing methods. The ability to produce lightweight parts, with sufficient mechanical properties, could be crucial in reducing fuel consumption and CO_2_ emissions, especially within aerospace applications. However, the mechanical and physical properties of thermoplastic polymers are heavily influenced by the crystallisation process and crystalline morphology [13]. This is of particular importance for critical end-use parts, such as aerospace components, where dimensional precision and mechanical performance are vital. During LS, crystallisation is associated with a volume reduction, which can affect the geometrical accuracy of the final part; non-uniform crystallisation within parts can result in warpage, rendering the component unserviceable [14]. Polyamide-12 (PA-12) is the most common commercial polymer within laser sintering, and a key characteristic of this material is a large processing window [2,4,5,6]. This ensures that there is a substantial difference between the crystallisation temperature (T_c_) and the melting temperature (T_m_), preventing crystallisation occurring during the build and reducing the shrinkage process. However, the crystallisation kinetics of PA-12 are also heavily dependent on multiple processing parameters, such as temperature, time, cooling rate, pressure, and melt orientation [7,15]. Furthermore, PA-12 is metastable and polymorphic, which can result in a multi-phase crystallisation process whereby, depending on temperature, PA-12 may transform into various different crystal phases [7]. From an industrial standpoint, understanding this behaviour is crucial because changes in the extent of crystallinity development, and type of crystalline morphology, can significantly affect the thermal and mechanical properties of a fabricated part [15].

Polymer crystallisation involves primary and secondary processes, which are often treated in isolation. Primary crystallisation is initiated when the polymer melt is cooled below T_m_, via a process known as nucleation. Homogenous nucleation is where disordered molecules, within entangled amorphous polymer chains, are able to rotate, allowing small nuclei to form spontaneously throughout the polymer melt [16]. However, nucleation can also occur heterogeneously, whereby impurities present in the polymer system act as artificial primary nuclei [16]. Following the formation of stable nuclei, free rotation of flexible polymer chains allows chain folding and alignment into a lamellar structure, whereby adjacent crystalline blocks are separated by a mobile, inter-lamellar amorphous phase [17]. This results in the formation of spherulites, which grow radially from the nuclei until spherulite impingement, indicating completion of the primary process. The rate of primary crystallisation is related to the degree of supercooling from the melt. At temperatures near the T_m_, crystal growth is thermodynamically favoured so nucleation is limited, resulting in a relatively smaller number of nuclei that grow into large spherulites. The presence of large spherulites increases the strength of the polymer but also renders the material brittle, emphasising how an understanding of crystallisation kinetics can allow close control of material morphology and, subsequently, the mechanical properties of LS parts.

Traditionally, it was considered that secondary crystallisation describes any further developments of crystallinity, which occur following impingement of spherulites, permitting previously “non-crystallisable units”, which were unable to contribute to primary crystallisation, to integrate into the growing lamellae structure [18]. This can refer to lateral thickening of the crystalline lamella formed during primary crystallisation [19,20,21] and the growth of new, smaller crystal structures within the amorphous fraction through “infilling” [15,19,20] (Figure S1). During primary crystallisation, radial growth of lamellae generally occurs linearly with time until spherulite impingement [22]; however, an alternative kinetic mechanism is required to define the secondary process. Conventionally, a logarithmic increase in secondary crystallisation with time was reported [23,24,25], but more recently, it has been suggested that secondary crystallisation adheres to a root time dependence, which relates to a diffusion-controlled process [18,20,21,22]. This theory was proposed by Hay, who also presented evidence for primary and secondary crystallisation occurring simultaneously, contradicting previous suggestions that the secondary process can only initiate following termination of primary crystallisation through spherulite impingement [26]. Lamellae produced in the early stages of crystallisation, at the centre of the spherulite, were observed to be thicker than at the outer boundary, indicating that secondary crystallisation must occur within the spherulite as soon as lamellae are formed [22,27].

Another important consideration of LS is the requirement for significant amounts of support material, resulting in 80–90% of feedstock powder remaining un-sintered during each build. Therefore, to ensure the economic and environmental sustainability of the process, un-sintered powder remaining in the build chamber must be re-used in future builds [11,28]. However, during each build, PA-12 is exposed to temperatures close to the material melting point for prolonged time periods, leading to aging and degradation, which affect the quality and re-usability of the material. At the powder bed temperature of ~170 °C, multiple aging processes, namely, polycondensation, secondary crystallisation, cross-linking, and chain scission, often occur concurrently [29,30,31]. To counteract the effect of these aging phenomena, it is common for the un-sintered (“used”) powder to be “refreshed” with virgin material before re-use in subsequent builds. As such, a refresh ratio is commonly used, which refers to the proportion of recycled to virgin powder, and typically ranges from 30–50% virgin powder [29,32]. Nonetheless, within the LS industry, there is currently a limited understanding regarding the effect of re-using PA-12 powder across multiple build cycles on the crystallisation kinetics of PA-12 powder.

Previous studies have shown that aging and degradation have the potential to alter the crystallisation behaviour, melt flowability, and particle coalescence of PA-12 powder. When polyamides are exposed to elevated temperatures, increased mobility of amorphous regions can result in increased crystallinity via secondary crystallisation [28,30,33]. Multiple papers have reported that successive powder re-use, or simulated oven-conditioning, causes a reduction in melt flow rate; this can be attributed to an increased cross-link density [9,30,33], or a rise in molecular weight (M_w_), as a result of polycondensation [4,5,7,9,11]. In the context of LS, reductions in melt flowability can limit full coalescence of PA-12 particles. As LS occurs under atmospheric pressure, coalescence is particularly important because particle viscosity and surface tension become the driving force for complete consolidation and compaction of the powder layer [34]. Therefore, reductions in material coalescence can hinder the surface finish of LS parts [34] and cause increased part porosity, which often results in embrittlement of fabricated components [32,35,36]. However, there have been no previous investigations into the effect of powder re-use, aging, and virgin refresh rates on the isothermal crystallisation kinetics of PA-12 powder. As crystallisation is so crucial to fabricating LS parts with sufficient mechanical and structural properties, this research is necessary to help reveal more information about the behaviour of un-sintered powder and its suitability for re-use in future builds.

There are multiple models that aim to best illustrate the isothermal crystallisation kinetics of polymers. The Avrami model undoubtedly remains the most popular isothermal crystallisation model and has been used frequently to explain the crystallisation kinetics of various polyamides [37,38,39], including LS grade PA-12 powder [7,14,31,40]. However, despite widespread use, the Avrami model has been criticised due to a number of limitations [41]. In practice, the Avrami model is only suitable for modelling primary crystallisation and is unable to accurately describe the latter stages of crystallisation that follow spherulite impingement. As such, Avrami often returns non-integer values for the Avrami exponent, which provides limited information regarding the mechanism for nucleation and growth [22,26,42]. The Avrami model also fails to account for an induction time, and differential scanning calorimetry (DSC) often returns incomplete exotherms, leading to integration errors and inaccurate data [41]. These limitations have led various authors to propose alternative models that aim to resolve these problems by modifying the existing Avrami equation. These include, but are not limited to, the simplified Hillier [43], Tobin [44], Malkin [45], and Hay models [20,21,46,47].

Previous work has begun to explore the isothermal crystallisation kinetics of PA-12 powder in the context of laser sintering [14,40,48]. A study from Neugebauer et al. explored both the isothermal and non-isothermal crystallisation kinetics of polyamide-12 used within LS [14]. This study intended to emulate the laser exposure found within the LS procedure. However, they used a cooling rate of only 40 °C/min to reach the target isothermal temperature, which is likely slower than the rate of cool-down that the powder experiences after laser scanning. Similarly, this rate may not be quick enough to prevent crystallisation initiating during cooling prior to the isothermal step. Zhao, Wudy, and Drummer explored the isothermal crystallisation kinetics of PA-12 between 160–168 °C, using a faster cooling rate of 60 °C/min. They observed that the Avrami model can only accurately fit 70% of the full crystallisation process; beyond this point, the experimental data deviate away from the modelled curve. However, their work was limited to the Avrami model alone, and the various alternative models were not considered [40]. Furthermore, these papers do not explore the effect of powder re-use, and subsequent material aging, on the isothermal crystallisation kinetics of PA-12.

Paolucci et al. investigated the influence of aging on the crystallisation kinetics of PA-12 powder [7]. Virgin PA-12 powder was stored at a range of temperatures for extended time periods, in a nitrogen flushed oven, to replicate the conditions of an LS build chamber. Thermal treatment resulted in polycondensation, indicated by increases in M_w_ and melt viscosity. Fast scanning calorimetry experiments on the “annealed” PA-12 samples suggested that powder aging does not affect crystal nucleation but causes a reduction in the crystal growth rate of PA-12. Nevertheless, it was reported that this reduction in crystallisation kinetics only occurred in a specific temperature range between 100–150 °C, which is below the expected powder bed temperature during LS. Also, oven storage cannot directly replicate complex powder re-use procedures, such as the use of refresh ratios, which restricts the industrial relevance of their work.

This paper investigates the coalescence behaviour, melt flowability, and sintering kinetics of PA-12 powder in order to identify the key aging processes occurring when feedstock material is re-used across multiple LS build cycles with a 70:30 refresh ratio. Via differential scanning calorimetry experiments, the effect of powder re-use, and subsequent aging processes, on the isothermal crystallisation kinetics of PA-12 is quantified and modelled using the traditional Avrami model. Furthermore, to the best of the authors knowledge, the applicability of alternative models, namely, simplified Hillier, Tobin, Malkin, and Hay, to describe the crystallisation behaviour of LS grade PA-12 powder is yet to be determined. As such, this work also uses non-linear regression analysis to determine the suitability of each of these models for describing the isothermal crystallisation behaviour of virgin and re-used (aged) PA-12 powder. Finally, the most appropriate model for analysing the crystallisation kinetics of virgin PA-12 powders, and PA-12 feedstock re-used in up to six LS build cycles, is proposed.

## 2. Experimental Method

Prototal UK Ltd. (Newbury, UK) provided industrial grade virgin PA-12 (PA2200) powder (EOS GmbH, Krailling, Germany) and refreshed PA-12 powder samples that had been re-used in up to 7 laser sintering builds, using a refresh ratio of 70:30 (used: virgin) for each build cycle. In this study, virgin powder and powder re-used in 2, 4, and 6 build cycles were analysed. These are referred to as used 2, used 4, and used 6, respectively.

### 2.1. Powder Characterisation

#### 2.1.1. Hot-Stage Microscopy (HSM)

The effect of powder re-use on the melting and coalescence behaviour of PA-12 powder was visualised using a Keyence 4K digital microscope (Keyence UK Ltd., Milton Keynes, UK), equipped with a Linkam THMS600 heating stage and a Linkam TMS 94 temperature controller (Linkam Scientific Instruments Ltd., Redhill, UK). To investigate the coalescence behaviour, virgin and re-used powders were heated from 25 °C to 220 °C at 10 °C/min, and samples were viewed using a microscope magnification of 300×. Alternatively, for the isothermal particle “sintering” experiment, samples were heated from 25 °C to the sintering temperature (195 °C, 200 °C, 205 °C) at 150 °C/min and held at that temperature for 5 min. In this case, to focus on two individual powder particles, the magnification was 1000×.

#### 2.1.2. Differential Scanning Calorimetry (DSC)—Isothermal Crystallisation

A Mettler Toledo differential scanning calorimeter, DSC 1 (Mettler Toledo, Schwerzenbach, Switzerland) calibrated with zinc (T_m_ 419.5 °C, ΔH_f_ 107.5 Jg^−1^) and indium (T_m_ 156.6 °C, ΔH_f_ 28.45 Jg^−1^), was used to determine the isothermal crystallisation kinetics of the PA-12 powder. Powder samples (6 ± 0.5 mg), were placed into Mettler Toledo 40 μL aluminium DSC pans, capped with aluminium lids, and sealed with a press. All experiments were conducted under a nitrogen flow rate of 100 cm^3^/min, and a pinhole was placed in the top of each DSC pan. A Huber TC100 immersion cooler (Huber Kaltemaschinenbau AG, Offenburg, Germany) provided temperature control for extended isothermal segments.

The DSC protocol involved isothermal experiments from the molten state, also known as melt-crystallisation. In each DSC experiment, samples were heated at 30 °C min^−1^ to 220 °C and held for 3 min to eliminate any residual crystals. They were then cooled at 70 °C/min to the designated isothermal T_c_ (162–169 °C) and held for up to 420 min. The cooling rate of 70 °C/min is the maximum capability of the DSC-1 system and was selected to try and prevent any crystallisation initiating on the cool-down. Following the isothermal hold, samples were immediately re-heated to 220 °C at 10 °C/min to analyse the effect of isothermal temperature on the polymers melt behaviour. The heat of fusion (ΔH_f_) was determined from the melting peak and used to calculate percentage crystallinity (X_c_) using Equation (1), whereby ΔH_f_^0^ = 209.3 Jg^−1^ (100% crystalline PA-12) [2].

(1) $$X_{c}\left(\%\right)=\frac{{\Delta H}_{f}}{{{\Delta H}_{f}}^{0}}\times 100$$

To investigate the isothermal crystallisation kinetics of virgin powder and material re-used in 2, 4, and 6 LS build cycles, raw data from the isothermal DSC segment was extracted and the change in heat flow, as a function of time, measured. These raw data, after being subjected to baseline and induction time corrections, were used to analyse and compare the Avrami [49], simplified Hillier [43,50], Tobin [44], and Malkin [45] models (Equations S1-S5). To enable visual comparison with the Hay model [22,51], cumulative crystallinity data were converted to true fractional crystallinity using Equation (2). For a detailed explanation of each model, refer to previous work from Kelly and Jenkins [15,47].

(2) $$Fractional\;crystallinity=\frac{Cumulative\;area\;under\;the\;exotherm\;(Wsg^{-1})}{Heat\;of\;fusion\;(Jg^{-1})}$$

Following the isothermal crystallisation experiments, the crystallisation kinetic parameters were estimated from traditional double log plots for the Avrami, simplified Hillier, Tobin, and Hay models, whilst the Malkin parameters were established from the Avrami results. Using SPSS software (IBM, Portsmouth, UK), the calculated parameters were then employed with each model to produce fractional crystallinity versus time plots. The fit of these plots was compared with experimental data and analysed using visual evaluation, as well as values for the coefficient of determination (R^2^) and standard error of regression, s (Equation (3)):

(3) $$s=\sqrt{\frac{\sum {\left(X_{t}-{X^{\prime }}_{t}\right)}^{2}}{n-2}},$$

where s is the standard error of regression, X_t_ is experimental fractional crystallinity, X^′^_t_ is the modelled fractional crystallinity, and *n* is the number of data points. Additionally, SPSS was utilised to generate curve fittings for each model using non-linear regression. This produced multivariable kinetic parameters, which were also used to produce fractional crystallinity curves and analysed via the same methods. These various statistical analysis techniques were applied to understand the applicability of each model as a function of aging state; as such, the most suitable model for both virgin and re-used PA-12 powder was determined.

## 3. Results and Discussion

### 3.1. The Effect of Powder Re-Use on Sintering and Coalescence Behaviour of PA-12

**Figure 1:**
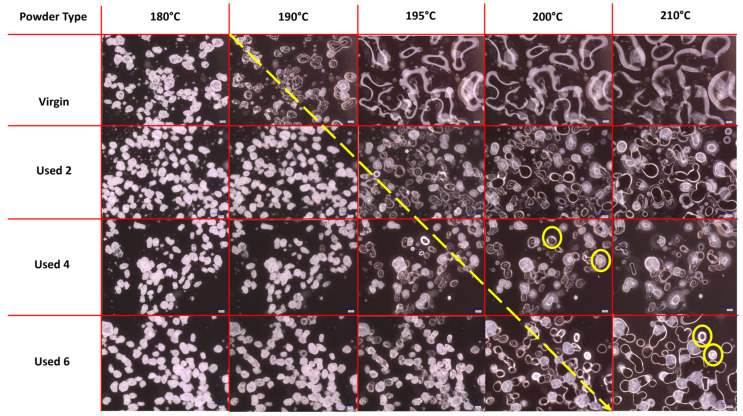
Powder melting and coalescence behaviour of virgin and used powder, observed using hot stage microscopy by heating samples at a constant heating rate of 10 °C/min. The theoretical yellow arrow indicates the change from particle softening to melting and coalescence during heating; yellow circles provide examples of incomplete melting and the presence of un-molten particle cores.

The melt flowability and coalescence behaviour of PA-12 powder is a useful indicator of powder quality because it can have a significant effect on the structural and mechanical properties of final LS parts. Nonetheless, the use of hot-stage microscopy for characterising recycled polymeric powder is less common than other techniques such as DSC or melt-flow index. In this study, HSM showed a reduction in melt flowability and material coalescence as a function of powder re-use (Figure 1). Upon heating, virgin powder particles remain solid until at least 180 °C; however by 190 °C, melting has already begun, and at 195 °C, the material is in an entirely molten state. Therefore, melting of virgin material occurs sharply with a moderate melting point range. The molten polymer can flow easily without restriction; by 210 °C, there is minimal free volume remaining within the sample, which represents an increased coalescence rate and good melt flowability. However, with increased powder re-use, melting initiates at progressively higher temperatures (indicated by the theoretical yellow arrow in Figure 1) and over a notably broader temperature range. This is further shown by DSC experiments, where an increased peak T_m_ and T_m_ range is observed as a function of build number (Figure S2). In used powder samples, consolidation of the molten material is limited, even at temperatures as high as 210 °C, suggesting a significant decrease in powder coalescence and melt flowability. Similarly, in used 4 and 6 powder samples, un-molten particle cores are present at 200 °C and 210 °C, respectively, indicating that increased powder re-use could lead to incomplete melting during the sintering process.

Successive re-use of PA-12 powder in LS builds could result in polycondensation, an aging process that causes macromolecular chain growth via reactions of free-chain end groups (Figure S3). Ultimately, this often results in a significant increase in M_w_ [2,6,8,28,52] and melt viscosity, which hinders the flowability of the molten phase [4,7,10,30,53]. For example, Sillani et al. calculated that the molecular weight of recycled PA-12 powder (50:50 refresh ratio) was 163% greater than virgin powder after only one build cycle [2]. Similarly, Wudy and Drummer used gel-permeation chromatography to show that the M_w_ of PA-12 powder almost doubles after re-use for five build cycles [28]. In both cases, the increase in M_w_ was attributed to the polycondensation process. Furthermore, storage of PA-12 powder at elevated temperatures during LS can cause intermolecular cross-linking, leading to the formation of “tie-chains” within the polymer structure (Figure S1). A tie chain represents a portion of a disordered molecule, which is able to traverse the free space between adjacent crystalline lamellae unobstructed [54]. This forms a connection between lamellae structures, restricting the mobility of the inter-lamellae amorphous chains, leading to increases in melt viscosity [9,32,33]. As a result, cross-linking may also contribute to the observed reduction in melt flow and particle coalescence.

**Figure 2:**
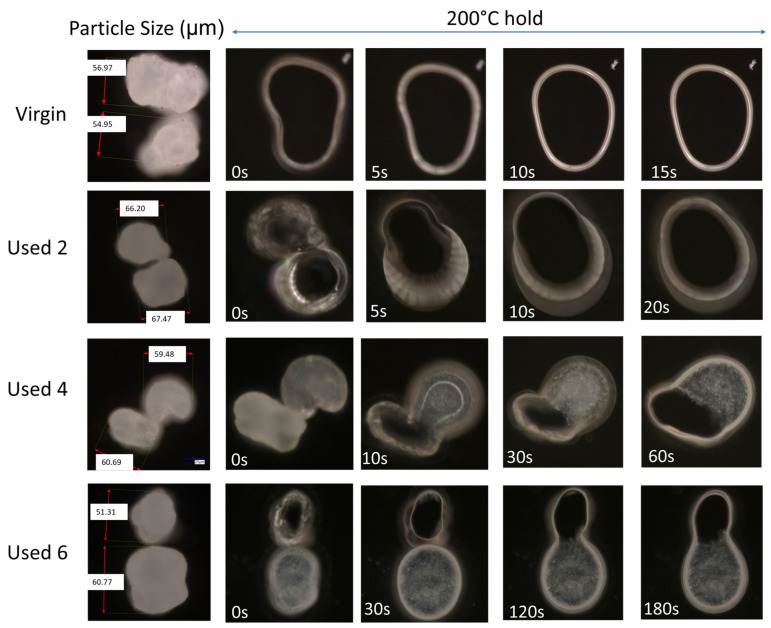
The coalescence behaviour, at 200 °C, of two virgin powder particles, and powder recovered from different LS build cycles.

**Figure 3:**
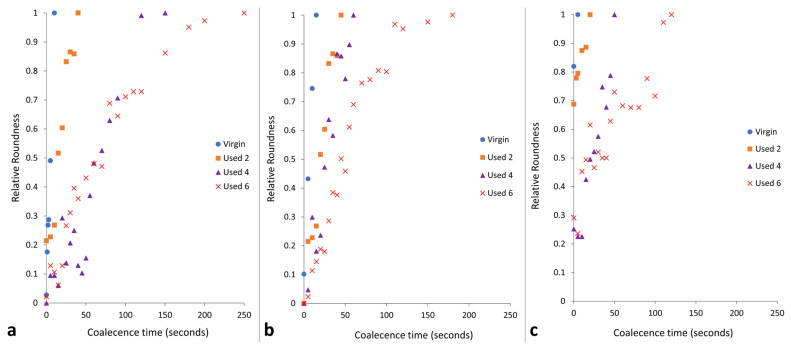
The relative roundness of two powder particles coalescing into one consolidated, melted ‘particle’ as a function of time when holding at coalescence temperatures of (**a**) 195 °C, (**b**) 200 °C, and (**c**) 205 °C.

The change in coalescence behaviour of PA-12 powder is also evident in Figure 2. In virgin material, at a constant ‘sintering’ temperature of 200 °C, two separate particles can combine to form one ‘fused’ particle within 10 s, exhibiting fast coalescence. However, in used powder, at the same temperature of 200 °C, particle coalescence is much slower. With increased powder re-use, a progressively higher thermal exposure time is required to ensure consolidation of the two particles, emphasising a reduction in the flowability and coalescence of the molten material. This experiment was also repeated at temperatures of 195 °C and 205 °C (Figure S4). The coalescence rate of PA-12 powders, as a function of powder re-use and isothermal temperature, was quantified using relative roundness, which describes the time it takes for two separate particles to merge into one “combined” particle (Figure 3). Relative roundness (Equation (4)) is measured using ImageJ and provides a qualitative, dimensionless value, whereby 0.0 relates to two separate particles and 1.0 correlates to a fully consolidated, single, ‘round’ particle.

(4) $$4\times \frac{\left[Area\right]}{\pi \times {\left[Major\;axis\right]}^{2}}$$

The data presented in Figure 3 demonstrate that the coalescence behaviour is heavily dependent on temperature. Independent of powder type, with increased hold temperature, the rate of particle coalescence increases. For example, at 205 °C, powder recovered from build 6 reaches a relative roundness of 1.0 after only 120 s, significantly faster than the 250 s required at a temperature of 195 °C for the same powder type. However, relative roundness is also affected by powder re-use. At all temperatures, the coalescence time for virgin powder is less than 15 s, but with increased build number, the time required for full particle coalescence progressively rises.

If the results shown in Figure 1, Figure 2 and Figure 3 are contextualised to the scale of a laser sintering build chamber, then the impact of reduced melt flowability and restricted coalescence become increasingly relevant. Assuming that build processing parameters remain constant, the higher melting points of used powder (Figure 1) may cause incomplete melting during the sintering process, resulting in the presence of un-molten particle cores [4,33]. In addition, the influence of temperature on the coalescence behaviour of virgin and re-used powder is significant when considering the layer-by-layer nature of the LS process. To ensure sufficient coalescence of particles within each layer, and vertically between layers, a high melt flowability is required. However, as the number of layers for a particular component progressively increases, the bottom layers of that part become further away from the heat-source, causing a decrease in the localised temperature. Similarly, it is possible for large temperature gradients to form within a LS build chamber [33,55]. Figure 3 demonstrated that reductions in temperature, and increased powder re-use, hinder the rate of particle coalescence. As such, there may be limited thermal energy, or insufficient time, for complete coalescence in the lower layers of the build chamber, which prevents full interlayer bonding and increases pore density within vertically orientated parts. This could contribute to the anisotropic behaviour often observed in LS parts, whereby vertically orientated samples have the weakest tensile strength and are the most brittle [53,56]. Overall, un-molten particles and reduced melt flow rate cause reduced material coalescence, resulting in surface defects (orange peel) [34] and increased porosity [32,35,57] within parts fabricated from re-used powder. Increased porosity reduces part strength and induces embrittlement [32,36], which could render LS parts un-fit for purpose, particularly in critical end-use applications.

### 3.2. The Effect of Powder Re-Use on Crystallisation Behaviour of PA-12

**Figure 4:**
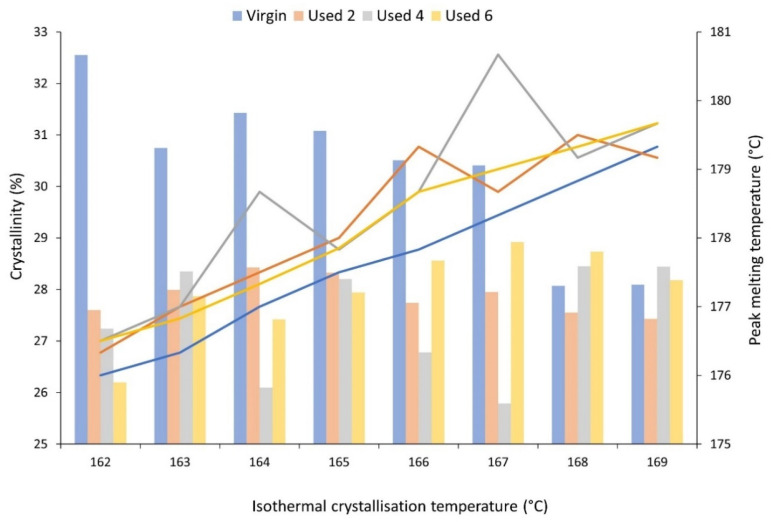
The change in absolute crystallinity (columns), measured from the exothermal crystallisation peak, and peak melting temperature (lines), measured on the subsequent re-heat, as a function of isothermal crystallisation temperature for virgin and used powder samples.

The mechanical and physical properties of LS parts are also heavily dependent on the crystallisation process and the crystalline morphology that develops. Therefore, isothermal crystallisation experiments were conducted using DSC to understand the effect of powder re-use and subsequent aging processes on the crystallisation behaviour of PA-12. During these experiments, values for absolute crystallinity, measured from the isothermal crystallisation exotherm and peak melting temperature, recorded on the immediate re-heat after the isothermal hold, were obtained (Figure 4). Independent of powder aging state, with increased isothermal T_c_, there is an increase in peak melting temperature because diffusion and growth dominate nucleation, so thicker and more perfect lamellae can develop [40,56]. More importantly, at each isothermal crystallisation temperature, used powder samples display a higher T_m_ than the respective virgin sample, indicating that powder re-use increases melting temperature. Previous literature suggests that an increase in the T_m_ of polyamides can be caused by polycondensation [11,30,53], secondary crystallisation [9,28,58], and cross-linking [30,59]. However, the increases in T_m_ observed in this experiment cannot be explained by secondary crystallisation because all traces of crystallinity are removed on the heating segment prior to the isothermal hold. On the other hand, although polycondensation is typically a reversible reaction, an LS build chamber combines high temperatures with an inert atmosphere, which forces the reaction towards continuous water removal. As such, under the conditions present during LS, un-sintered PA-12 powder is effectively exposed to a non-reversible polycondensation reaction. Cross-linking is also irreversible, so both of these processes cause permanent structural changes to PA-12. As such, polycondensation and cross-linking appear to be the dominant cause for the observed increase in T_m_ with successive powder re-use, and this may have significant consequences for the quality of final LS parts, as explained in Section 3.1.

Additionally, for isothermal T_c_’s between 162–167 °C, crystallinity development is higher within virgin powder samples than re-used material, which provides further evidence for the occurrence of polycondensation and cross-linking during LS (Figure 4). These aging processes result in structural defects, which reduce chain mobility and prevent amorphous fractions from re-ordering. This hinders the crystallisation process, resulting in reduced crystallinity development within used powder samples during isothermal crystallisation. The reported values for crystallinity were calculated using the enthalpy of crystallisation (ΔH_c_) from the isothermal crystallisation exotherm. To ensure that no reorganisation processes were occurring during re-heating, enthalpy of fusion (ΔH_f_) was also measured from the melting endotherm, and, within the limits of experimental variability, the change in ΔH_f_ is equal to the change in ΔH_c_ (Figure S5). This confirms that the observed changes in thermal properties and crystallisation behaviour, as a function of powder re-use, were a result of aging rather than crystal reorganisation during re-heating. Furthermore, the most significant increase in T_m_, and the largest reduction in crystallinity, occurs over the first two build cycles (Figure 4). For example, at an isothermal T_c_ of 162 °C, there is a 3.45% reduction in absolute crystallinity between virgin powder and material recovered from build 2; similarly, T_m_ increases by 0.9 °C. However, these changes are followed by a plateau, whereby the difference across the next four build cycles is minimal. It has been suggested previously that polycondensation occurs more rapidly in thermally unstressed material, i.e., virgin PA-12 powder; in used, aged powder, further chain growth is hindered by a reduced availability of end groups [6,12,30,57].

**Figure 5:**
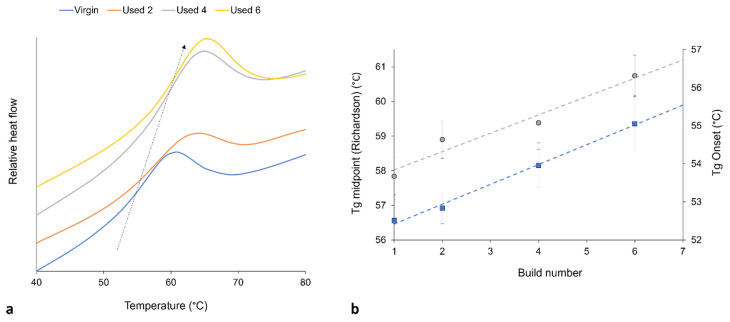
(**a**) With increased powder re-use, there is a shift in T_g_ to higher temperatures (represented by arrow), as recorded by DSC; (**b**) shows the increase in T_g_ onset (blue datapoints) and T_g_ midpoint (gray datapoints), whereby T_g_ is measured using the Richardson approach, and the plotted values are taken as an average from 10 repeats.

It is generally agreed that the glass transition temperature (T_g_) of linear polymers is dependent upon the average M_w_ of the system; polymers with a greater M_w_ usually have a higher T_g_ [60,61,62,63]. This relationship is related to the free-volume present in the system, whereby a reduction in free volume limits the mobility of amorphous chains, therefore causing an increase in T_g_ [54,60]. Polycondensation [2,6,8,28,52] and cross-linking [9,30,32] are both thought to increase the M_w_ of PA-12. These aging processes also cause an increase in chain-end and cross-link density, which decreases the free-volume present in the inter-lamellae amorphous phase. With increased powder re-use, there is an increase in the onset and midpoint of T_g_, thus suggesting an increase in M_w_ (Figure 5). This provides further evidence of polycondensation and cross-linking occurring with successive re-use of PA-12 within LS.

### 3.3. Avrami Analysis of Isothermal Crystallisation

**Figure 6:**
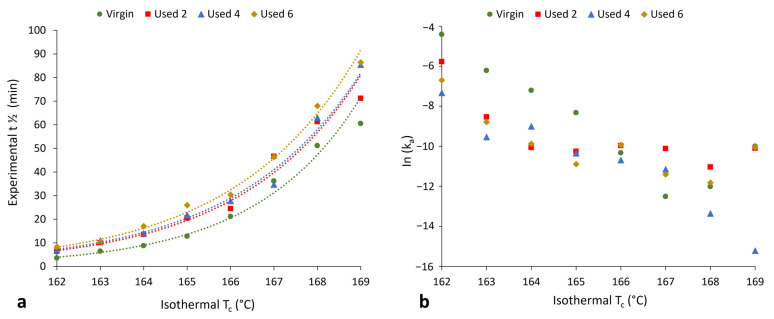
The change in (**a**) crystallisation half-life and (**b**) Avrami rate constant (k_a_) as a function of isothermal crystallisation temperature for each powder type.

Section 3.1 and Section 3.2 have provided evidence that shows polycondensation and cross-linking are the key aging processes occurring when PA-12 is recycled across multiple LS builds. In addition to hindering material coalescence, reducing melt flowability, and decreasing the extent of crystallinity development, these processes also influence the rate of isothermal crystallisation of PA-12. Avrami is the most commonly used kinetic model for describing the crystallisation behaviour of polymeric materials. However, the effect of powder re-use, and related aging processes, on the applicability of the Avrami model is yet to be determined. Therefore, isothermal crystallisation experiments were conducted, for each powder type, in the temperature range of 162–169 °C, and the raw data are presented in Figure S6. In both virgin and used powder, as the T_c_ increases, there is a reduction in the rate of crystallisation, as reported previously [14,40,48]. Using the Avrami model, the raw DSC data were converted into double log plots, whereby the experimental data are limited to primary crystallisation alone (Figure S7). Data are restricted to the linear region so that the Avrami crystallisation kinetic parameters, n and k, can be accurately estimated from the gradient and y-intercept, respectively. The estimated kinetic parameters are displayed in Table S1. These data were used to create the plots presented in Figure 6, highlighting that with increased powder re-use, there is a reduction in the crystallisation kinetics of PA-12. Used powder was found to have a greater crystallisation half-life (t ½) than virgin material (Figure 6a), indicating a reduction in the rate of crystallisation due to morphological changes caused by polycondensation and cross-linking. Chain entanglements, knots, and permanent, non-reversible tie-chains create disorder and steric hindrance, reducing the number of stable nuclei, which can form within the polymer system. These structural changes also restrict chain mobility and prevent chains from re-ordering into a crystalline structure. As such, the rate of both nucleation and growth decreases, reducing the crystallisation kinetics and overall extent of crystallinity development. The reduction in crystallisation rate is further shown in Figure 6b, which, particularly for lower isothermal T_c_’s, displays a reduction in the Avrami rate constant, k_a_.

**Figure 7:**
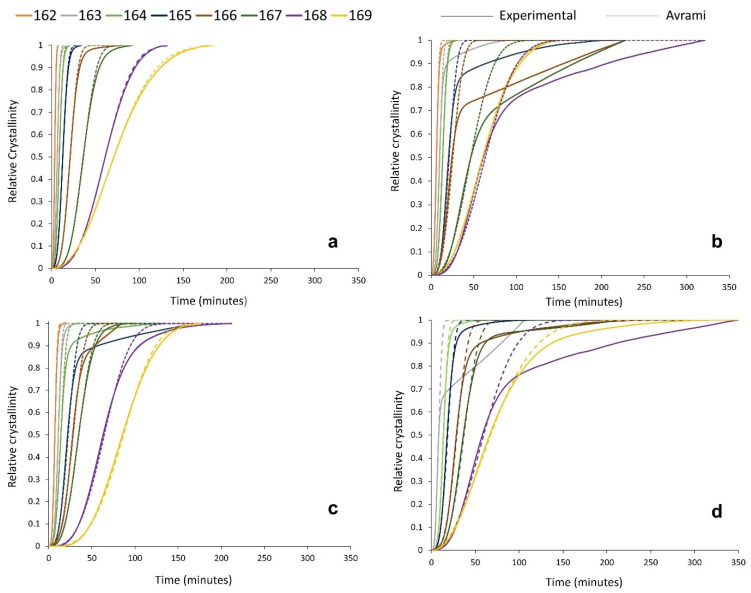
Relative crystallinity vs. time curves, created from both experimental data and the Avrami model within SPSS for (**a**) virgin powder and used powder collected from build (**b**) 2, (**c**) 4, and (**d**) 6.

To better understand the effect of powder re-use on the crystallisation behaviour of PA-12 powder at every isothermal crystallisation temperature, the kinetic parameters (n_a_ and k_a_) presented in Table S1 were applied to create multiple relative cumulative crystallinity curves using the Avrami equation (Figure 7). These plots track the progression of phase transformation as a function of time for each powder type, providing information about the crystallisation process and the fit of the Avrami model. In virgin powder, at every crystallisation temperature, the experimental data form a typical sigmoidal curve. Initially, there is a crystallisation induction time, followed by accelerated primary crystallisation at a constant rate before retardation of the crystallisation process. In the latter stages of crystallisation, the rate of change in relative crystallinity with time is very small, and a pseudo-equilibrium level of crystallinity is obtained, which represents completion of the process [54]. In virgin material, the Avrami model almost parallels the experimental data (Figure 7a), and the constant, linear increase in crystallinity, as a function of time, continues until an almost complete phase transformation. This illustrates that the crystallisation behaviour of virgin PA-12 powder adheres to the Avrami model, and termination proceeds via spherulite impingement. This is further supported by co-efficient of determination (R^2^) values of >0.995 for the virgin samples (Table S2).

However, the used powder experimental data deviate away from the Avrami model at significantly lower levels of phase transformation. This is most clearly visualised in Figure 7b, whereby, at multiple isothermal T_c_’s, used powder recovered from build 2 only conforms to the Avrami theory for up to ~60–70% of transformation. Beyond this point, there are considerable deviations, and the crystallisation rate reduces significantly. A similar trend is observed in used powder recovered from builds 4 and 6; however, the difference between the experimental data and the Avrami model is most significant with used 2 powder. This is also reflected by the R^2^ values for used material (Table S2), which are substantially lower than virgin samples, indicating that the Avrami theory can only accurately model a portion of the crystallisation process within aged powder.

Previous work from Mandelkern et al. suggests that deviations from the Avrami expression occur as M_w_ increases [54,64]. The influence of M_w_ may explain why, within re-used powder, the Avrami expression can accurately explain the early stages of crystallisation but fails as phase transformation progresses. The observation that Avrami is unable to fit the experimental data at higher levels of phase transformation indicates that the crystallisation process is no longer obliging to the Avrami theory of termination via spherulite impingement [54]. Instead, other factors, such as microstructural defects, must become involved as crystallisation progresses. This paper has provided significant evidence to suggest that polycondensation and cross-linking occur when PA-12 powder is re-used across multiple LS build cycles. Both processes cause an increase in M_w_, which alters the morphology and microstructure of the polymer; therefore, they are the most likely cause for the observed deviation from the Avrami model in used powder samples. Polycondensation and the lengthening of non-crystalline amorphous polymer chains causes an increase in the concentration of permanent structural defects, such as chain entanglements and knots, which are unable to participate in the crystallisation process. Similarly, intermolecular cross-linking results in tie-chains, and these structures cannot be reversed or dissipated during melting or crystallisation. Therefore, there are fewer crystallisable units available within the polymer system, and the rate of nucleation and growth cannot continue at the assumed constant rate. Therefore, at this stage, there is no longer a linear increase in relative crystallinity as a function of time, so the progression of primary crystallisation is impeded [54], causing the experimental data to deviate away from the Avrami model at earlier stages of phase transformation. Furthermore, the level of deviation is most significant in powder recovered from build 2, which can be explained by the suggestion that polycondensation occurs more readily within thermally unstressed material. This supports the crystallisation behaviour displayed in Figure 4, whereby the largest change in crystallinity development and T_m_ occurred over the first two build cycles, before a plateau was observed.

### 3.4. Modelling the Crystallisation Kinetics of Re-Used PA-12 Powder

**Figure 8:**
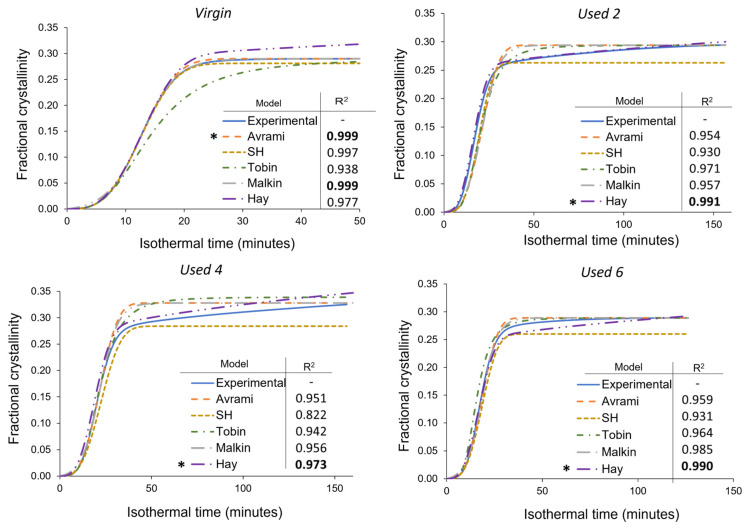
Comparison of the Avrami, simplified Hillier, Tobin, Malkin, and Hay models with experimental data obtained for PA-12 at 165 °C for each powder type. Fractional crystallinity curves were fabricated by inputting the crystallisation kinetic parameters (Tables S3–S5) into the corresponding models. Superimposed on each plot is a key and the respective co-efficient of determination (R^2^) value for each model, * represents the model with the highest R^2^ in each plot.

Although the most commonly used model for analysing the isothermal crystallisation kinetics of polymers, the Avrami theory can only accurately model a portion of the crystallisation process within aged material. Consequently, Avrami is unsuitable for modelling the overall crystallisation behaviour of re-used PA-12 powder, and a more appropriate model is required for used, aged powder. As explained in Section 2.1.2, the Avrami, simplified Hillier, Tobin, and Hay crystallisation kinetic parameters were obtained from traditional double log plots, whilst the Malkin parameters were calculated from Avrami data (Tables S3–S5). To investigate the suitability of each kinetic model as a function of powder re-use, SPSS software determined the fit of these kinetic parameters to the experimental data for each powder type at a constant crystallisation temperature of 165 °C (Figure 8). In this case, all cumulative data were converted to actual fractional crystallinity using Equation (2). A temperature of 165 °C was selected because it is in the middle of the crystallisation temperature range studied, so there is a compromise in terms of crystallisation rate. This ensures that crystallisation does not occur too rapidly, yet fast enough that the small changes in heat flow, with time, can still be consistently measured accurately. Nonetheless, similar behaviour was observed at every isothermal crystallisation temperature.

In virgin powder, the Avrami model sufficiently describes the full crystallisation process, emphasised by an R^2^ value of 0.999, whilst the simplified Hillier and Malkin models also produce high R^2^ values. Since virgin powder has not been exposed to aging processes, the experimental data do not deviate away from the constant primary crystallisation rate assumed within the Avrami model. Conversely, the Hay and Tobin models have lower R^2^ values and are unable to accurately track the whole crystallisation process. Therefore, the traditional Avrami theory is the most suitable model for describing the crystallisation behaviour of virgin LS-grade PA-12 powder. On the other hand, Figure 8 emphasises that Avrami is unable to precisely model the full phase transformation for re-used PA-12 powder, supporting the results shown in Figure 7. The Avrami equation sufficiently defines primary crystallisation; however, it cannot accurately track the region where the rate of change in fractional crystallinity significantly reduces. As such, relative to virgin PA-12 powder, the Avrami model has substantially lower R^2^ values for re-used powder.

**Figure 9:**
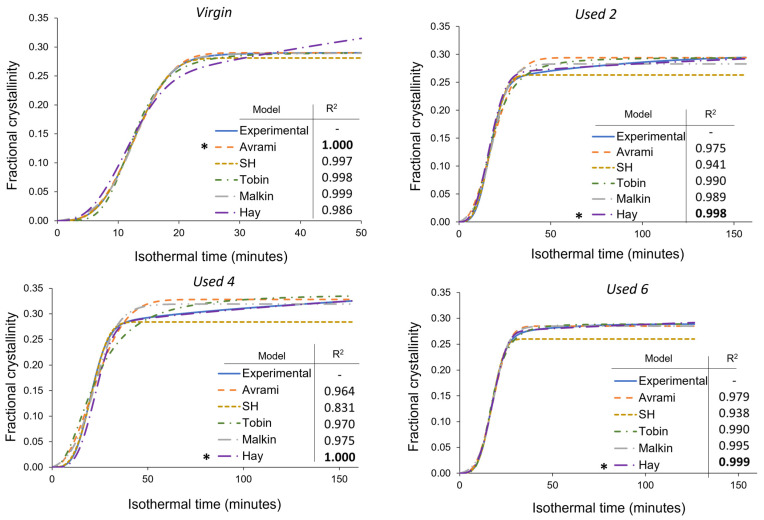
Nonlinear multivariable curve fitting of the Avrami, simplified Hillier, Tobin, Malkin, and Hay kinetic models to the isothermal crystallisation of PA-12 at 165 °C for each powder type. Superimposed on each plot is a key and the respective co-efficient of determination (R^2^) value for each model,* represents the model with the highest R^2^ in each plot.

As mentioned in Section 3.3, deviations away from the Avrami model, within used powder, occur when primary crystallisation becomes limited due to restricted mobility of long, entangled, and knotted polymer chains. As such, re-used powder contains more “non-crystallisable units” that are unable to contribute to the primary process, so the rate and extent of primary crystallisation reduces. Therefore, for full phase transformation to occur, a greater contribution from secondary crystallisation is required. It is thought that secondary crystallisation is a thickening process that adheres to either a log [23,24,25] or a root time [18,47] dependence, so it occurs relatively slowly compared to primary crystallisation. For the secondary process to occur, rearrangement of relatively immobile, highly entangled inter-lamellar amorphous regions is required. Additionally, polycondensdation and cross-linking also reduce the rate of secondary crystallisation as lamellar thickening becomes restricted by diffusion of structural defects away from the lamellar growth front [21]; this further increases the time required for full phase transformation within re-used powder samples. Hay suggested that the overlap of primary and secondary crystallisation explains the non-integer n values commonly observed when using the Avrami equation in polymer crystallisation [22,51]. Hay also presented evidence to demonstrate that primary and secondary processes occur simultaneously [22,27,51], so the Hay model is designed to include contributions from both primary and secondary crystallisation throughout the entire curve. As a result, Hay’s model is the most effective for describing the full crystallisation behaviour of used powder because it can accurately estimate the latter stages of crystallisation when the contribution from the secondary process is more significant. This is emphasised by the high R^2^ values reported when modelling the isothermal crystallisation of used 2, used 4, and used 6 datasets with the Hay theory (Figure 8). Using SPSS software, the experimental data were also curve-fitted to each respective model via nonlinear, multivariable regression analysis (Figure 9). These curves revealed almost identical results and similar R^2^ values for both virgin and re-used powder. This further demonstrates that although the Avrami model is suitable for modelling the behaviour of virgin PA-12 powder, the Hay model is most appropriate for describing the full crystallisation process within re-used, aged material.

A more comprehensive approach for comparing different models is to consider the standard error of regression, s, (Equation (3)) for four individual areas of the cumulative fractional crystallinity curve, whereby the lower the *s* value, the better the fit between the model and experimental data. These four regions resemble the initial stages of crystallisation, the region dominated by primary crystallisation, a transition segment, and the secondary crystallisation region. Other than the initial stage (0 < X_t_ < 0.1), the exact X_t_ values for each region varies depending on powder type. This method of analysis allows the suitability of each model to be compared at different stages of the crystallisation process. The data presented in Table 1 show that within virgin powder, all models have low s values for the initial and primary regions of crystallisation, suggesting that each model can accurately describe the crystallisation induction period and primary crystallisation process. However, only the Avrami and Malkin models could successfully model the transition and secondary crystallisation regions. This is because in virgin powder, primary crystallisation dominates, and there is limited involvement of the secondary crystallisation process. The Hay model, for example, expects a rise in fractional crystallinity due to secondary crystallisation; in reality, as the primary process is not limited by hindered chain mobility, almost full phase transformation occurs without much input from the secondary process. As such, the s values shown in Table 1 emphasise that the Avrami (and Malkin) models provide the closest fit to the experimental data for each stage of the crystallisation process in virgin powder.

On the other hand, in re-used PA-12 powder, the Hay model generally has the lowest s values, especially in the transition and secondary crystallisation regions of the fractional crystallinity curve. Compared to virgin material, all models are less successful at tracking primary crystallisation. In virgin powder, the standard error of regression for the primary region was commonly calculated to be less than 0.002, whilst in re-used powder samples, s values are usually greater than 0.01. The higher s values are particularly noticeable in powder recovered from builds 2 and 4. In used powder, polycondensation and cross-linking reduce the rate of primary crystallisation, resulting in an inconsistent growth rate. Therefore, predicting and describing the change in fractional crystallinity as a function of time becomes more difficult. These aging processes also limit the extent of the primary crystallisation process, causing the experimental data to deviate away from the Avrami model. As such, the s values in the transition and secondary regions are also generally greater within used powder than the respective values in virgin samples (Table 1).

The Avrami model is unable to accurately describe the latter stages of phase transformation in re-used powder because Avrami assumes that the rate of crystal growth is linear and constant. However, structural defects caused by polycondensation and cross-linking reduce the availability of crystallisable polymer units; therefore, the rate of nucleation and growth are no longer constant, with respect to the extent of phase transformation [54]. The simplified Hillier, Tobin, and Malkin models are derived from, and closely related to, Avrami and therefore display similar results. Hay is the only model that accounts for primary and secondary crystallisation simultaneously. Similarly, Hay does not presume a constant nucleation and growth rate, so it is better suited to describe the behaviour of aged PA-12 powder, whereby structural defects hinder the crystallisation rate in the latter stages of phase transformation. As such, within re-used powder samples, Hay is considerably more successful at modelling the transition and secondary crystallisation portion of the curves, and s values are typically 0.005 or less.

These results, calculated for an isothermal crystallisation temperature of 165 °C, are highlighted by the data presented in Tables S6 and S7, which show that comparable results were obtained at every isothermal T_c_. In these data, there is a focus on the Avrami and Hay models because they were the two most successful models for describing the crystallisation behaviour of virgin and used powder, respectively. Table S6 illustrates that for virgin powder, the Avrami model has the highest R^2^ values, whilst the Hay model has higher R^2^ values for every batch of re-used powder. This is further shown by Table S7, which compares the s values of virgin and re-used powder at each isothermal T_c_ for the Avrami and Hay models. At 168 °C and 169 °C, datapoints are missing for the Hay model. This theory is unsuitable at higher temperatures because the crystallisation rate becomes so slow that extended isothermal times of >8 h are required to fully model the whole crystallisation process. It was observed that the DSC is unable to precisely maintain temperature over this extended time period or accurately monitor the small changes in heat-flow. Nonetheless, 162–167 °C is a sufficient temperature range to demonstrate the advantages of using the Hay model to investigate the isothermal crystallisation of aged PA-12 powder.

**Figure 10:**
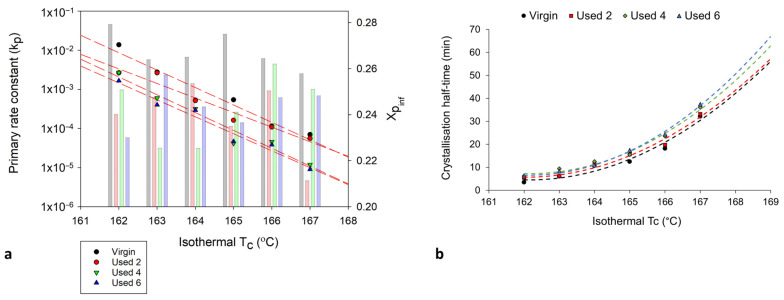
The change in (**a**) the primary crystallisation rate constant, k_p_ (datapoints) and Xp_inf_ (columns) and (**b**) the crystallisation half-time, whereby the trendline is extrapolated to include isothermal T_c_’s: 168 °C and 169 °C, calculated using the Hay model, as a function of isothermal T_c_ and powder type.

**Figure 11:**
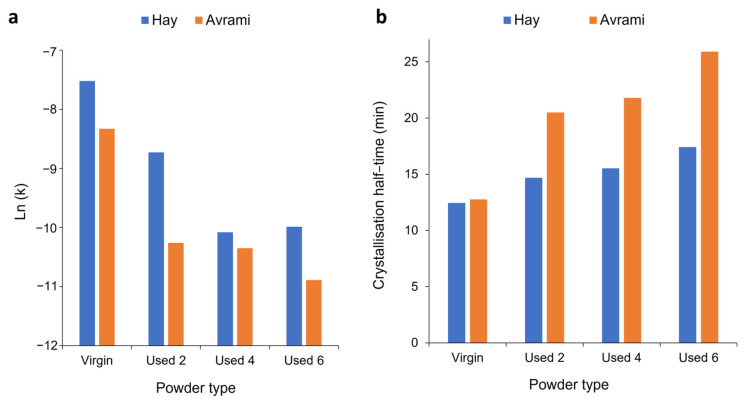
Comparison of the values of (**a**) k_p_ and (**b**) t ½ using both the Hay and Avrami models, for each powder type, at an isothermal T_c_ of 165 °C.

To fully understand the suitability of the Hay model for describing the crystallisation behaviour of used powder, it is necessary to explore the relationship between powder type and Hay’s kinetic parameters. Crystallisation half-time (t ½) can also be estimated using the Hay model, whereby half-time is taken to be the time at which $X_{t}=\frac{X_{p,inf}}{2}$ [51], whereby X_p,inf_ describes the final fractional crystallinity upon completion of the Avrami primary process. Figure 10 demonstrates that with an increased isothermal T_c_, and increased powder re-use, there is generally a reduction in Hay’s primary rate constant (k_p_) and Hay’s t ½. These same trends were observed when using the Avrami model to calculate k_a_ and t ½ (Figure 6), and the similarity between the Hay and Avrami estimations is shown in Figure 11. Therefore, independent of the kinetic model employed, it is clear that there is a reduction in the primary crystallisation rate as a function of powder re-use. The Hay model offers additional, unique parameters that can reveal more information about the full crystallisation process following the primary region. Although there does not appear to be a significant change in the secondary rate constant (k_s_), as a function of powder re-use (Table S8), a reduction in X_p,inf_ is observed (Figure 10a). This shows that primary crystallisation terminates at lower levels of phase transformation within re-used powder, further supporting the relative crystallinity curves presented in Figure 7. Values of X_p,inf_ are also important when considering the estimations of crystallisation half-time using the Hay method. The extent of the increase in t ½, as a function of powder re-use, is modest when calculated using Hay compared to Avrami (Figure 11b). In virgin material, the values for t ½ are almost identical; however, in used powder, Hay calculates t ½ to be lower. This occurs because Hay’s estimation of t ½ only includes the primary crystallisation process, and with increased powder re-use, the extent of the primary process is reduced, leading to lower values for t ½. Overall, the similarity between the values of the kinetic parameters for Avrami and Hay emphasises that both models can successfully model the primary region of crystallisation for all powder types. However, Hay has the unique advantage of being able to estimate the point at which the primary process terminates and can successfully model the secondary crystallisation region. Further comparisons of the Hay and Avrami kinetic parameters, at every isothermal T_c_, are provided in Table S8.

The results outlined in this section have emphasised that although the traditional Avrami model is the most suitable for describing the crystallisation behaviour of virgin LS grade PA-12 powder, it is unsuitable and inaccurate for powder that has been re-used in multiple LS builds. However, the Hay model can sufficiently describe the complete crystallisation process for re-used PA-12 powder. To the best of the author’s knowledge, this is the first study to establish the most appropriate kinetic model for describing the crystallisation behaviour of re-used and aged PA-12.

## 4. Conclusions

In this study, PA-12 powder was re-used for a total of seven laser sintering (LS) build cycles, using a 70:30 refresh ratio. Hot-stage microscopy indicated that as a function of powder re-use, there is an increase in melting temperature, a reduction in melt flowability, and restricted particle coalescence. These changes in the thermal behaviour of PA-12, attributed to polycondensation and cross-linking, can cause increased porosity and reduced mechanical properties within LS parts fabricated from re-used powder. The mechanical and structural properties of final parts are also heavily dependent on the crystallisation behaviour of the polymer. In this work, the isothermal crystallisation of PA-12 was studied using a range of models, namely, Avrami, simplified Hillier, Tobin, Malkin, and Hay. For virgin material, the Avrami model was found to be the most successful at modelling the entire crystallisation process. However, in re-used powder, polycondensation and cross-linking cause structural defects that reduce the rate and extent of primary crystallisation, limiting the applicability of the Avrami model. Nonetheless, as a result of accounting for both primary and secondary crystallisation, the Hay model accurately describes the whole crystallisation process and is therefore the optimum method for defining the crystallisation behaviour of aged polymeric material. From an industry perspective, applying the Hay model to better understand the crystallisation behaviour of re-used PA-12 powder could help ensure the fabrication of LS parts with sufficient mechanical properties and a high dimensional precision.

## Figures and Tables

**Table 1:** Standard error of regression for each isothermal crystallisation kinetic model over four selected regions of the cumulative fractional crystallinity curve.

Powder Type	Region	X_t_ Range	Avrami		Simplified Hillier		Tobin		Malkin		Hay	
			DLP	CF	DLP	CF	DLP	CF	DLP	CF	DLP	CF
Virgin	Initial	0.0–0.1	0.0003	0.0011	0.0003	0.0003	0.0004	0.0052	0.0044	0.0034	0.0009	0.0058
	Primary	0.1–0.8	0.0010	0.0020	0.0016	0.0004	0.0286	0.0064	0.0018	0.0017	0.0020	0.0109
	Transition	0.8–0.95	0.0053	0.0020	0.0013	0.0008	0.0527	0.0049	0.0007	0.0012	0.0101	0.0173
	Secondary	0.95–1.0	0.0024	0.0019	0.0077	0.0077	0.0232	0.0032	0.0006	0.0005	0.0212	0.0135
Used 2	Initial	0.0–0.1	0.002	0.010	0.003	0.002	0.003	0.005	0.002	0.008	0.002	0.006
	Primary	0.1–0.7	0.037	0.012	0.029	0.002	0.032	0.010	0.037	0.007	0.011	0.011
	Transition	0.7–0.9	0.026	0.013	0.009	0.002	0.020	0.010	0.023	0.009	0.009	0.007
	Secondary	0.9–1.0	0.013	0.013	0.022	0.022	0.010	0.008	0.013	0.008	0.002	0.003
Used 4	Initial	0.0–0.1	0.0012	0.0149	0.0008	0.0016	0.0004	0.0201	0.0058	0.0123	0.0076	0.0019
	Primary	0.1–0.7	0.0121	0.0186	0.0257	0.0018	0.0041	0.0240	0.0045	0.0113	0.0225	0.0023
	Transition	0.7–0.9	0.0293	0.0226	0.0140	0.0058	0.0254	0.0213	0.0317	0.0173	0.0108	0.0014
	Secondary	0.9–1.0	0.0172	0.0169	0.0309	0.0309	0.0253	0.0136	0.0172	0.0107	0.0163	0.0006
Used 6	Initial	0.0–0.1	0.0008	0.0035	0.0005	0.0004	0.0048	0.0054	0.0055	0.0010	0.0018	0.0015
	Primary	0.1–0.8	0.0061	0.0051	0.0193	0.0012	0.0062	0.0038	0.0324	0.0035	0.0023	0.0012
	Transition	0.8–0.95	0.0125	0.0073	0.0157	0.0081	0.0075	0.0055	0.0083	0.0047	0.0012	0.0004
	Secondary	0.95–1.0	0.0050	0.0036	0.0258	0.0258	0.0046	0.0033	0.0031	0.0021	0.0014	0.0005

## Data Availability

Data are contained within the article.

## Supplementary Materials

The following supporting information can be downloaded at: https://www.mdpi.com/article/10.3390/polym16050612/s1: Figure S1: Schematic diagram displaying different mechanisms of secondary crystallisation and cross-linking: (a) lamellar thickening, (b) lamellar infilling, and (c) tie-chain formation as a result of cross-linking. Equations (S1)–(S5): Crystallisation kinetics models. Figure S2: The change in (a) peak T_m_ and (b) T_m_ range with increased build number, whereby each datapoint is taken as an average of 3 repeats. Figure S3: Schematic diagram representing the polycondensation process, emphasising the entangled, knotted chain structures present in re-used powder. Figure S4: The coalescence behaviour of two virgin powder particles, and powder recovered from different LS build cycles at (a) 195 °C and (b) 205 °C. Figure S5: The change in enthalpy of crystallisation, measured from the exothermic peak during the isothermal segment (column) and enthalpy of fusion, measured from the endothermic melting peak, when re-heating at 10 °C/min (dotted line), as a function of isothermal crystallisation temperature, and powder re-use. Figure S6: Raw data from the DSC displaying the change in the shape and position of the exothermic crystallisation peak, as a function of isothermal crystallisation temperature for (a) virgin, (b) used 2, (c) used 4, and d) used 6 powder types. Figure S7: Avrami double log plots for each powder type, in each case the Xt data range was restricted to the linear region to ensure an R^2^ > 0.99. Table S1: Avrami crystallisation kinetic parameters and coefficients of determination (R^2^) derived from double log plots over iso crystallisation range of 162–169 °C. Xt range restricted to ensure a R^2^ value of > 0.99. Table S2: Co-efficient of determination (R^2^) values, calculated using SPSS software, for each isothermal crystallisation temperature and powder type. Table S3: Crystallisation kinetic parameters derived from double log plots (n and k) and from non-linear multi-variable regression analysis in SPSS (n* and k*). Table S4: A comparison of the kinetic parameters derived from double log plots (n and k) with the kinetic parameters derived from non-linear multi-variable regression analysis in SPSS (n* and k*) for the Hay model. Table S5: A comparison of the kinetic parameters derived from double log plots (n and k) with the kinetic parameters derived from non-linear multi-variable regression analysis in SPSS (n* and k*) for the Malkin model. Table S6: Co-efficient of determination (R^2^) values for the Avrami and Hay models at every isothermal crystallisation temperature. DLP curve is produced from parameters calculated through double log plots, CF curve is produced via curve fitting in SPSS. Table S7: Standard error of regression, s, values for the Avrami and Hay models, at every isothermal crystallisation temperature. DLP curve is produced from parameters calculated through double log plots, CF curve is produced via curve fitting in SPSS. Table S8: The change in kinetic parameters, as a function of powder re-use, calculated using the Hay and Avrami models.
