# Supplementary material for: The Effect of Powder Re-Use on the Coalescence Behaviour and Isothermal Crystallisation Kinetics of Polyamide 12 within Powder Bed Fusion
Source: Polymers (Basel). 2024 Feb 23;16(5):612. doi: 10.3390/polym16050612 (PMC10934940; doi:10.3390/polym16050612)
Supplement: Supplementary file 1 [file polymers-16-00612-s001.zip › polymers-2849770-supplementary.pdf]

Supplementary Information

Figure S1: Schematic diagram displaying different mechanisms of secondary crystallisation and cross-linking: a) lamellar thickening, b) lamellar infilling, and c) tie-chain formation as a result of cross-linking.

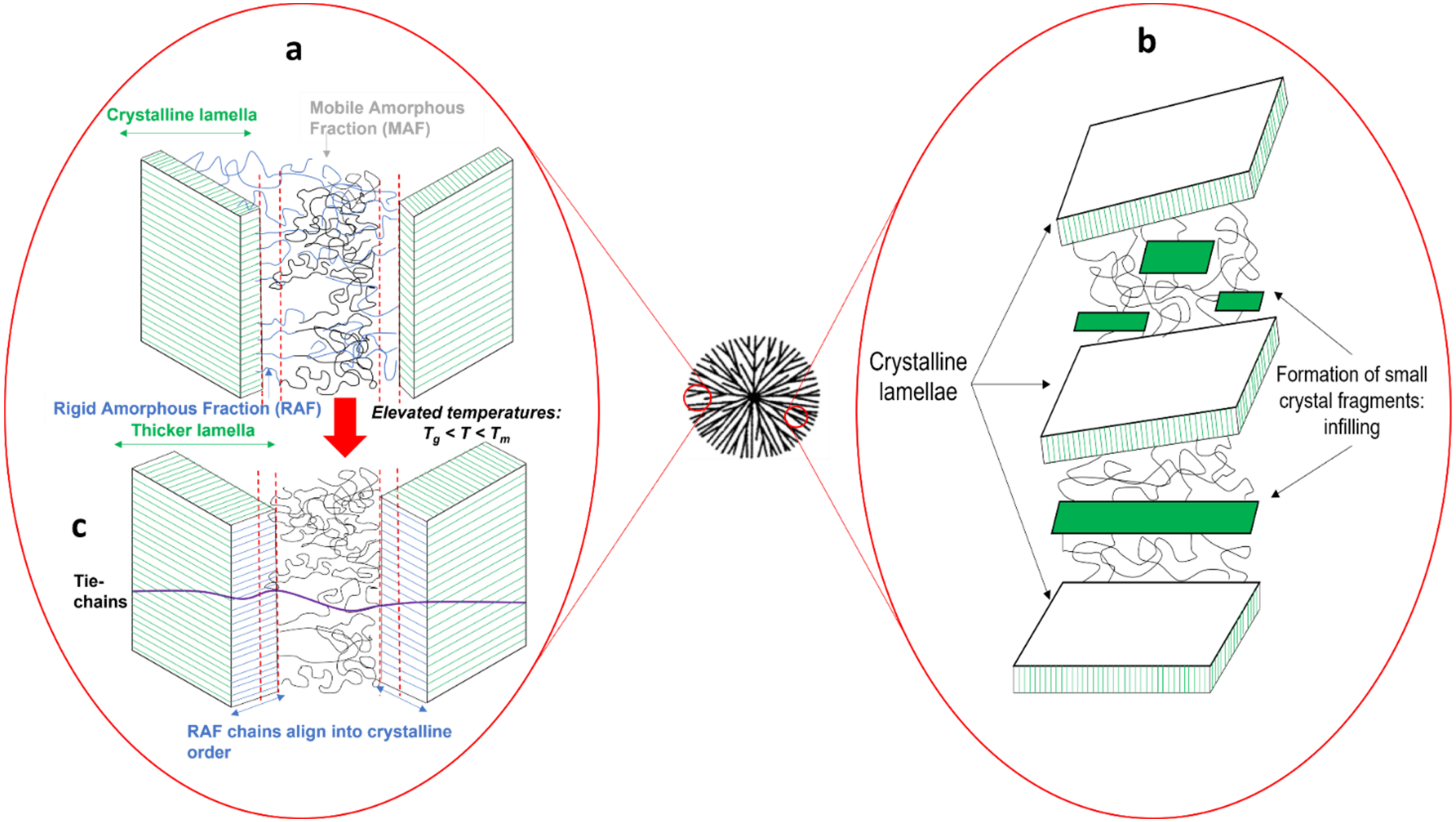

Equations S1-S5: Crystallisation kinetics models

Avrami:

$$X_t = \left(1 - e^{-k_a t^{n_a}}\right)$$

Where X_t is fractional crystallinity at time, t; k_a is the Avrami rate constant; and n_a is the Avrami exponent.

Simplified Hilllier:

$$X_t = X_{p,\infty}\left(1 - e^{-k_a t^{n_a}}\right)$$

Where X_t is relative crystallinity at time, t; $X_{p,\infty}$ is the relative crystallinity upon completion of primary crystallisation; k_a is the Avrami rate constant; and n_a is the Avrami exponent.

Tobin:

$$X_t = \frac{k_t t^{n_t}}{1+(k_t t^{n_t})}$$

Where X_t is fractional crystallinity at time, t; k_t is the Tobin rate constant; whilst n_t is the Tobin exponent.

Malkin:

$$X_t = 1 - \frac{C_0+1}{C_0+e^{C_1 t}}$$

Where C_0 and C_1 are defined as the Malkin exponent and Malkin crystallisation rate constant, respectively, and they are estimated using *Eqs. S5-S6.*

$$C_0 = 4^{n_a} - 4$$

$$C_1 = ln(4^{n_a} - 2)\left(\frac{k_a}{ln\,(2)}\right)^{\frac{1}{n_a}}$$

Hay

$$X_t = X_{p,\infty}\left(1 - e^{-k_p t^{n_a}}\right)\left(1 + k_s t^{1/2}\right)$$

Where X_t and $X_{p,\infty}$ are the fractional crystallinities at time, t, and conclusion of primary crystallisation, respectively; k_p and k_s are the Avrami rate constants of primary and secondary crystallisation, respectively; and n_a is the Avrami exponent.

Figure S2: The change in a) peak T_m and b) T_m range with increased build number, whereby each datapoint is taken as an average of 3 repeats.

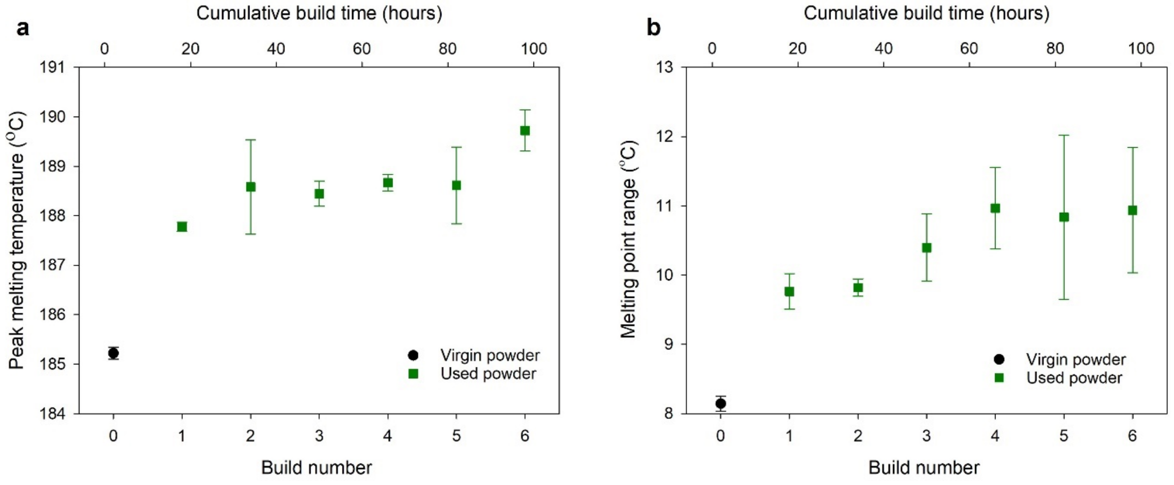

Figure S3: Schematic diagram representing the polycondensation process, emphasising the entangled, knotted chain structures present in re-used powder.

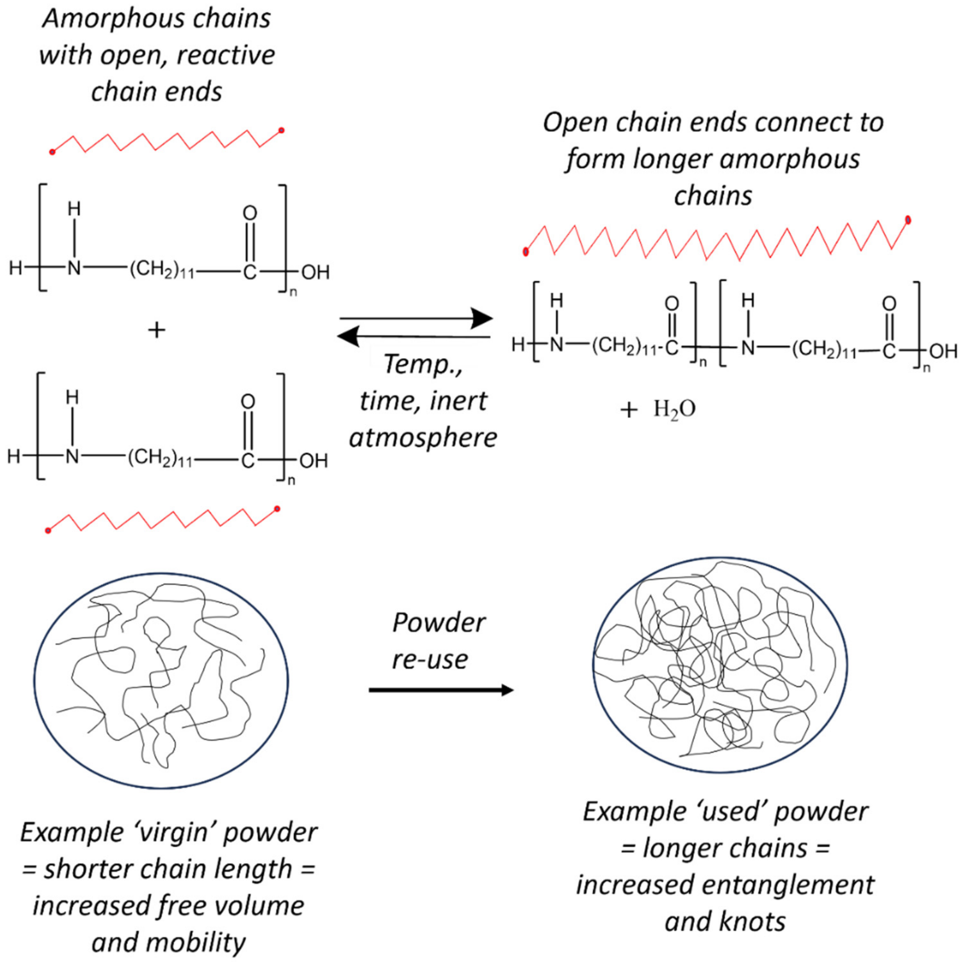

Figure S4: The coalescence behaviour of two virgin powder particles, and powder recovered from different LS build cycles at a) 195 °C and b) 205 °C.

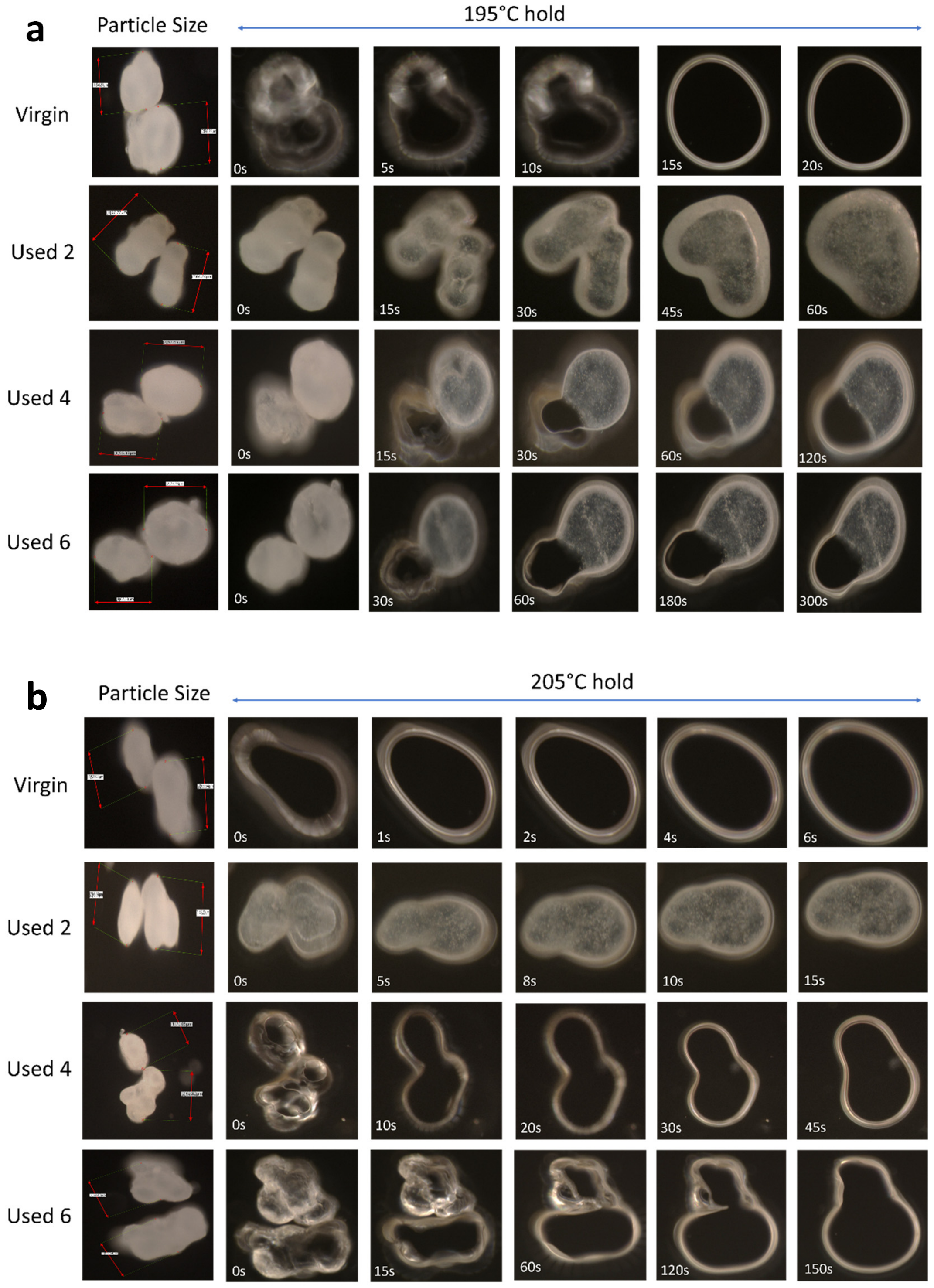

Figure S5: The change in enthalpy of crystallisation, measured from the exothermic peak during the isothermal segment (column) and enthalpy of fusion, measured from the endothermic melting peak, when re-heating at 10 °C min^{-1} (dotted line), as a function of isothermal crystallisation temperature, and powder re-use.

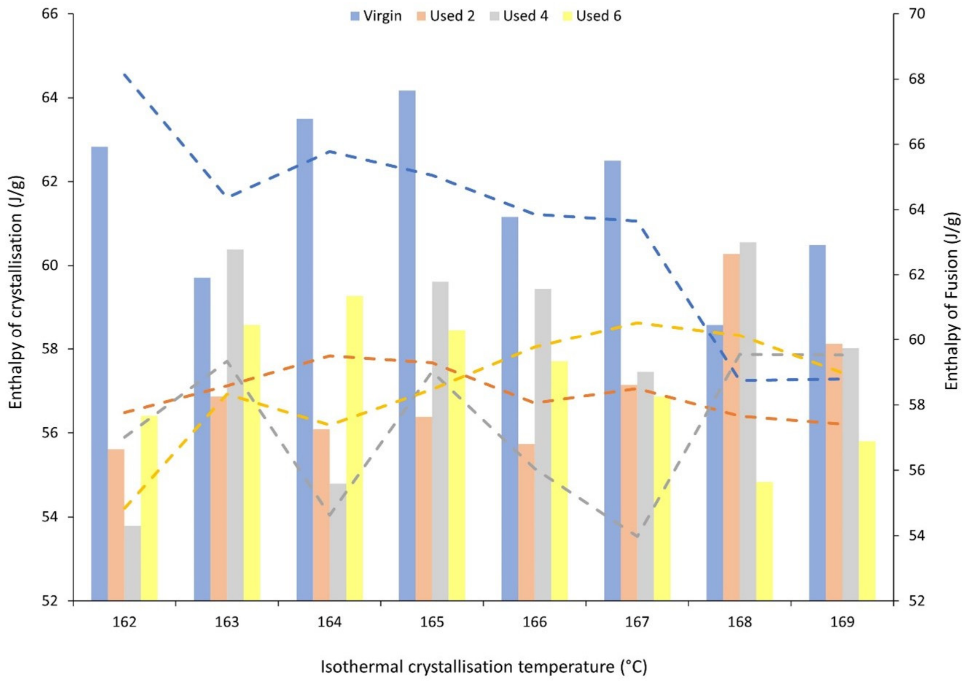

Figure S6: Raw data from the DSC displaying the change in the shape and position of the exothermic crystallisation peak, as a function of isothermal crystallisation temperature for a) virgin, b) used 2, c) used 4, and d) used 6 powder types.

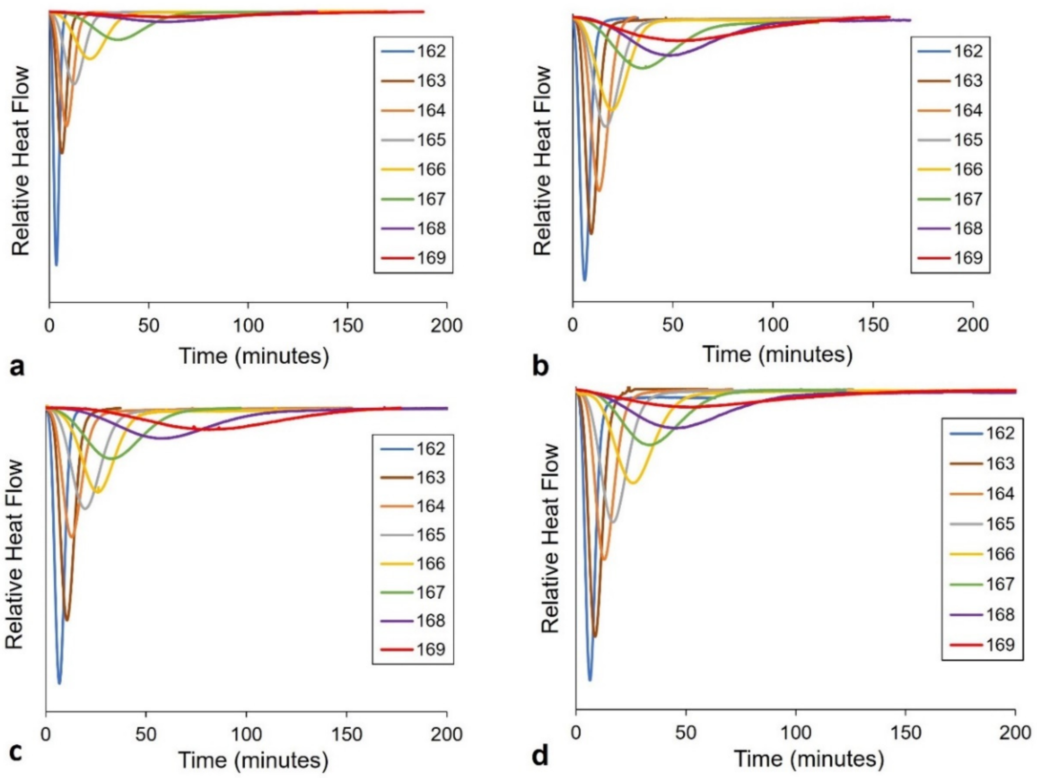

Figure S7: Avrami double log plots for each powder type, in each case the X_t data range was restricted to the linear region to ensure an R_2 > 0.99.

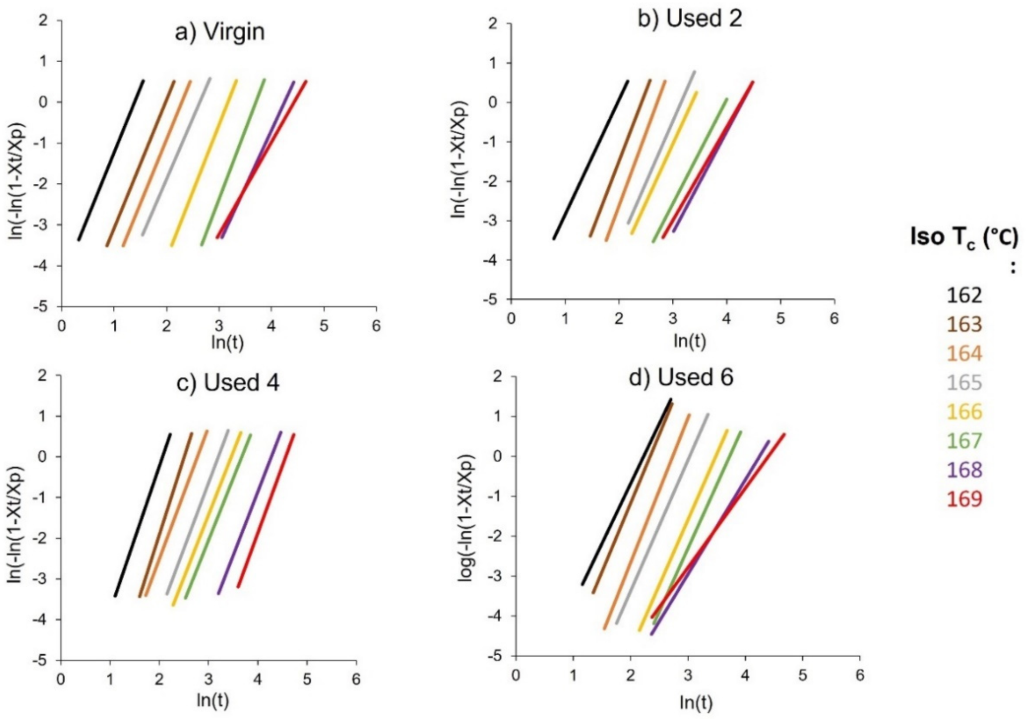

Table S1 - Avrami crystallisation kinetic parameters and coefficients of determination (R^2) derived from double log plots over iso crystallisation range of 162 – 169 °C. X_t range restricted to ensure a R^2 value of > 0.99.

Iso T_c	Virgin					Used 2				
	t ½ (min)	n_a	k_ax10^{-3} (min^{-n})	R^2	X_t range	t ½ (min)	n_a	k_ax10^{-3} (min^{-n})	R^2	X_t range
162	3.57	3.18	12.061	0.9997	0.03-0.95	6.35	2.93	3.085	0.9997	0.03-0.80
163	6.45	3.15	1.982	0.9999	0.03-0.95	10.18	3.52	0.196	0.9959	0.03-0.80
164	8.80	3.15	0.738	0.9999	0.03-0.95	13.60	3.71	0.043	0.9997	0.03-0.80
165	12.78	3.12	0.242	0.9999	0.03-0.95	21.18	3.24	0.035	0.9991	0.03-0.80
166	21.23	3.26	0.033	0.9998	0.03-0.95	25.43	2.97	0.046	0.9935	0.03-0.65
167	36.25	3.38	0.004	0.9995	0.03-0.95	46.56	2.54	0.040	0.9901	0.03-0.65
168	51.15	2.83	0.006	0.9992	0.03-0.95	63.57	2.57	0.016	0.9900	0.03-0.70
169	61.10	2.26	0.046	0.9997	0.03-0.95	71.25	2.37	0.041	0.9998	0.03-0.80

Iso T_c	Used 4					Used 6				
	t ½ (min)	n_a	k_ax10^{-3} (min^{-n})	R^2	X_t range	t ½ (min)	n_a	k_ax10^{-3} (min^{-n})	R^2	X_t range
162	7.10	3.55	0.652	0.9992	0.03-0.80	8.33	3.01	0.231	0.9912	0.07-0.55
163	11.10	3.81	0.072	0.9988	0.03-0.80	10.17	2.66	0.317	0.9992	0.03-0.90
164	14.10	3.25	0.123	0.9970	0.03-0.80	14.57	3.62	0.0511	0.9973	0.03-0.90
165	22.50	3.24	0.032	0.9965	0.03-0.80	23.95	3.28	0.0485	0.9951	0.03-0.90
166	28.25	3.09	0.023	0.9979	0.03-0.80	28.38	3.28	0.0112	0.9970	0.03-0.80
167	34.80	3.04	0.014	0.9996	0.03-0.80	36.38	3.17	0.0074	0.9977	0.03-0.80
168	64.40	3.12	0.002	0.9976	0.03-0.80	58.00	2.37	0.0432	0.9908	0.05-0.70
169	86.52	3.33	0.0002	0.9986	0.03-0.80	76.38	2.00	0.0153	0.9975	0.1-0.80

Table S2 - Co-efficient of determination (R^2) values, calculated using SPSS software, for each isothermal crystallisation temperature and powder type.

Isothermal T_c (°C)	SPSS Avrami R^2 values			
	Virgin	Used 2	Used 4	Used 6
162	0.999	0.997	0.999	0.234
163	0.997	0.976	0.894	0.953
164	0.998	0.999	0.979	0.997
165	0.999	0.954	0.951	0.959
166	0.999	0.912	0.795	0.875
167	0.995	0.817	0.899	0.897
168	1.000	0.818	0.988	0.887
169	0.999	0.985	0.903	0.993

Table S3: Crystallisation kinetic parameters derived from double log plots (n and k) and from non-linear multi-variable regression analysis in SPSS (n and k*).*

Powder type	Model	n	n*	Difference in n	k ($x10^{-3}$)	k* ($x10^{-3}$)	R^2
Virgin	Avrami	3.12	2.99	0.13	0.24	0.33	1
	SH	3.11	3.16	-0.05	0.257	0.23	0.997
	Tobin	3.77	4.62	-0.85	0.073	0.0085	0.998
Used 2	Avrami	3.24	2.19	1.05	0.035	1.133	0.975
	SH	3.56	3.01	0.55	0.018	0.13	0.941
	Tobin	4.03	3.06	1.03	0.0051	0.14	0.990
Used 4	Avrami	3.24	2.10	1.14	0.032	0.98	0.964
	SH	3.08	3.26	-0.12	0.047	0.37	0.831
	Tobin	3.88	2.26	1.62	0.0064	0.87	0.970
Used 6	Avrami	3.28	3.03	0.25	0.0485	0.0108	0.969
	SH	3.42	3.56	0.14	0.0422	0.0273	0.938
	Tobin	4.19	4.10	0.09	0.00573	0.00584	0.990

Table S4: A comparison of the kinetic parameters derived from double log plots (n and k) with the kinetic parameters derived from non-linear multi-variable regression analysis in SPSS (n and k*) for the Hay model*

Powder type	n	n*	Difference in n	k_p (x10^{-3}) (min^{-n})	k_p* (x10^{-3}) (min^{-n})	k_s (min^{-n})	k_s* (min^{-n})	R^2
Virgin	3.00	2.62	0.38	0.33	1.28	0.0283	0.1393	0.986
Used 2	3.00	2.75	0.25	0.16	0.31	0.0221	0.1381	0.998
Used 4	3.00	3.19	-0.19	0.11	0.047	0.0348	0.0250	1.000
Used 6	3.00	3.23	-0.23	0.15	0.0654	0.0525	0.0110	0.999

Table S5: A comparison of the kinetic parameters derived from double log plots (n and k) with the kinetic parameters derived from non-linear multi-variable regression analysis in SPSS (n and k*) for the Malkin model*

Powder Type	C_0	C_0*	Difference in C_0	C_1	C_1*	Difference in C_1	R^2
Virgin	71.80	83.23	-11.43	0.336	0.346	-0.01	1.000
Used 2	84.73	39.39	45.34	0.210	0.204	0.006	0.989
Used 4	84.93	32.84	52.09	0.204	0.159	0.045	0.975
Used 6	90.35	87.53	2.82	0.245	0.248	0.003	0.997

Table S6: Co-efficient of determination (R^2) values for the Avrami and Hay models at every isothermal crystallisation temperature. DLP curve is produced from parameters calculated through double log plots, CF curve is produced via curve fitting in SPSS.

	**Virgin**				**Used 2**				**Used 4**				**Used 6**			
Iso Tc	Avrami		Hay		Avrami		Hay		Avrami		Hay		Avrami		Hay	
	DLP	CF	DLP	CF	DLP	CF	DLP	CF	DLP	CF	DLP	CF	DLP	CF	DLP	CF
162	0.999	1.000	1.000	1.000	0.997	0.998	0.986	0.999	0.999	0.999	1.000	0.999	0.953	0.996	1.000	0.999
163	0.997	0.998	1.000	1.000	0.976	0.980	0.996	1.000	0.894	0.949	0.989	1.000	0.234	0.894	0.974	0.996
164	0.998	0.999	0.812	0.997	0.999	1.000	0.961	0.996	0.979	0.984	1.000	1.000	0.997	0.999	0.993	1.000
165	0.999	1.000	0.977	0.986	0.954	0.975	0.991	0.998	0.951	0.964	0.973	1.000	0.959	0.969	0.999	0.999
166	0.999	0.999	0.992	1.000	0.912	0.947	0.979	0.997	0.795	0.856	0.901	0.922	0.875	0.931	0.999	0.999
167	0.995	0.999	1.000	0.999	0.817	0.971	0.882	0.999	0.899	0.954	0.990	0.997	0.897	0.917	0.970	0.975
168	1.000	1.000	-	-	0.818	0.963	-	-	0.988	0.998	-	-	0.887	0.979	-	-
169	0.999	1.000	-	-	0.985	0.992	-	-	0.903	0.993	-	-	0.993	0.988	-	-

Table S7: Standard error of regression, s, values for the Avrami and Hay models, at every isothermal crystallisation temperature. DLP curve is produced from parameters calculated through double log plots, CF curve is produced via curve fitting in SPSS.

Iso Tc	*Region*	**Virgin**				**Used 2**				**Used 4**				**Used 6**			
		Avrami		Hay		Avrami		Hay		Avrami		Hay		Avrami		Hay	
		DLP	CF	DLP	CF	DLP	CF	DLP	CF	DLP	CF	DLP	CF	DLP	CF	DLP	CF
162	*Initial*	0.0004	0.0014	0.0010	0.0010	0.0003	0.0022	0.0003	0.0009	0.0002	0.0056	0.0006	0.0007	0.0190	0.0022	0.0016	0.0068
	Primary	0.0022	0.0015	0.0015	0.0010	0.0019	0.0034	0.0061	0.0013	0.0026	0.0952	0.0011	0.0014	0.0469	0.0032	0.0025	0.0104
	Transition	0.0034	0.0009	0.0018	0.0009	0.0093	0.0038	0.0093	0.0008	0.0083	0.1567	0.0044	0.0017	0.0198	0.0036	0.0034	0.0029
	Secondary	0.0027	0.0015	0.0002	0.0004	0.0043	0.0038	0.0080	0.0004	0.0031	0.1027	0.0010	0.0007	0.0031	0.0020	0.0007	0.0060
163	*Initial*	0.0006	0.0020	0.0009	0.0020	0.0007	0.0082	0.0046	0.0104	0.0003	0.0029	0.0023	0.0023	0.0016	0.0436	0.0131	0.0044
	Primary	0.0047	0.0035	0.0017	0.0043	0.0063	0.0113	0.0139	0.1445	0.0061	0.0572	0.0064	0.0018	0.0946	0.0291	0.0347	0.0045
	Transition	0.0116	0.0041	0.0011	0.0010	0.0216	0.0180	0.0054	0.2592	0.0125	0.1609	0.0009	0.0009	0.0528	0.0152	0.0092	0.0010
	Secondary	0.0046	0.0043	0.0000	0.0012	0.0760	0.0760	0.0004	0.2750	0.0043	0.0875	0.0006	0.0004	0.0115	0.0362	0.0007	0.0010
164	*Initial*	0.0005	0.0014	0.0088	0.0047	0.0006	0.0015	0.0001	0.0001	0.0007	0.0168	0.0008	0.0013	0.0006	0.0031	0.0085	0.0016
	Primary	0.0039	0.0026	0.0890	0.0088	0.0020	0.0025	0.0026	0.0006	0.0061	0.0572	0.0011	0.0014	0.0051	0.0044	0.0212	0.0022
	Transition	0.0091	0.0027	0.0545	0.0066	0.0076	0.0026	0.0049	0.0031	0.0206	0.0579	0.0002	0.0006	0.0113	0.0058	0.0034	0.0015
	Secondary	0.0037	0.0032	0.0002	0.0029	0.0033	0.0022	0.0192	0.0038	0.0061	0.1111	0.0002	0.0001	0.0040	0.0029	0.0001	0.0006
165	*Initial*	0.0003	0.0011	0.0009	0.0058	0.0020	0.0100	0.0035	0.0057	0.0012	0.0149	0.0076	0.0019	0.0008	0.0035	0.0018	0.0015
	Primary	0.0010	0.0020	0.0020	0.0109	0.0370	0.0120	0.0114	0.0106	0.0121	0.0186	0.0213	0.0021	0.0061	0.0051	0.0023	0.0012
	Transition	0.0053	0.0020	0.0100	0.0173	0.0260	0.0130	0.0090	0.0070	0.0293	0.0226	0.0093	0.0015	0.0125	0.0073	0.0012	0.0004
	Secondary	0.0024	0.0019	0.0212	0.0135	0.0130	0.0130	0.0024	0.0026	0.0172	0.0169	0.0163	0.0006	0.0050	0.0036	0.0014	0.0005
166	*Initial*	0.0006	0.0022	0.0038	0.0011	0.0037	0.0256	0.0005	0.0005	0.0041	0.0421	0.0025	0.0003	0.0015	0.0062	0.0031	0.0048
	Primary	0.0033	0.0038	0.0228	0.0011	0.0318	0.0281	0.0085	0.0005	0.0699	0.0282	0.0145	0.0233	0.0068	0.0097	0.0044	0.0076
	Transition	0.0115	0.0044	0.0047	0.0008	0.0313	0.0207	0.0220	0.0006	0.0189	0.0094	0.0850	0.0987	0.0263	0.0130	0.0016	0.0018

	Secondary	0.0044	0.0040	0.0002	0.0002	0.0910	0.0910	0.0314	0.0002	0.0040	0.0026	0.0112	0.0022	0.0080	0.0069	0.0021	0.0023
167	*Initial*	0.0013	0.0026	0.0020	0.0059	0.0020	0.0359	0.0083	0.0040	0.0001	0.0017	0.0004	0.0011	0.0004	0.0035	0.0146	0.0022
	Primary	0.0094	0.0040	0.0028	0.0094	0.0649	0.0241	0.0421	0.0008	0.0021	0.0023	0.0011	0.0020	0.0049	0.0042	0.0098	0.0010
	Transition	0.0161	0.0043	0.0005	0.0010	0.0534	0.0139	0.0524	0.0002	0.0063	0.0022	0.0015	0.0007	0.0086	0.0047	0.0657	0.0761
	Secondary	0.0049	0.0039	0.0006	0.0010	0.0116	0.0040	0.0565	0.0003	0.0300	0.0300	0.0025	0.0007	0.0170	0.0063	0.0023	0.0004
168	*Initial*	0.0005	0.0002	-	-	0.0023	0.0433	-	-	0.0031	0.0047	-	-	0.0025	0.0216	-	-
	Primary	0.0010	0.0004	-	-	0.0638	0.0291	-	-	0.0180	0.0057	-	-	0.0383	0.0181	-	-
	Transition	0.0014	0.0005	-	-	0.0529	0.0144	-	-	0.0227	0.0067	-	-	0.0477	0.0136	-	-
	Secondary	0.0006	0.0007	-	-	0.0121	0.0055	-	-	0.0041	0.0033	-	-	0.0113	0.0170	-	-
169	*Initial*	0.0002	0.0003	-	-	0.0030	0.0022	-	-	0.0017	0.0007	-	-	0.0014	0.0033	-	-
	Primary	0.0014	0.0006	-	-	0.0210	0.0312	-	-	0.0176	0.0032	-	-	0.0026	0.0031	-	-
	Transition	0.0027	0.0007	-	-	0.0370	0.0298	-	-	0.0088	0.0051	-	-	0.0100	0.0031	-	-
	Secondary	0.0009	0.0003	-	-	0.100	0.0981	-	-	0.0020	0.0019	-	-	0.0052	0.0029	-	-

Table S8: The change in kinetic parameters, as a function of powder re-use, calculated using the Hay and Avrami models.

Powder Type	Isothermal T_c (°C)	Hay				Avrami	
		Xp_{inf}	k_s	k_p	t ½ (min)	k_p	t ½ (min)
Virgin	162	0.279	0.0784	0.013801	3.50	0.012061	3.57
	163	0.264	0.0489	0.002760	6.05	0.001983	6.43
	164	0.265	0.0298	0.000530	10.75	0.000738	8.80
	165	0.275	0.0283	0.0005420	12.44	0.000242	12.76
	166	0.264	0.0242	0.0001150	18.20	0.000033	21.17
	167	0.258	0.0433	0.000070	32.15	0.000004	36.13
	168	-	-	-	-	0.000006	51.13
	169	-	-	-	-	0.000046	60.52
Used 2	162	0.240	0.0254	0.002661	5.48	0.003085	6.32
	163	0.248	0.0178	0.002650	6.22	0.000197	9.98
	164	0.254	0.0036	0.000516	11.31	0.000043	13.55
	165	0.235	0.0221	0.000162	13.68	0.000035	20.50
	166	0.250	0.0084	0.000109	19.76	0.000047	24.47
	167	0.211	0.0608	0.000056	33.2	0.000040	46.63
	168	-	-	-	-	0.000016	61.23
	169	-	-	-	-	0.000041	71.18
Used 4	162	0.251	0.0120	0.002670	6.25	0.000652	7.05
	163	0.226	0.0874	0.000597	9.40	0.000072	11.00
	164	0.225	0.0895	0.000320	12.52	0.000123	14.10
	165	0.241	0.0348	0.000042	15.53	0.000032	21.80
	166	0.262	0.0191	0.000046	23.58	0.000023	27.75
	167	0.251	0.0245	0.000012	36.10	0.000014	34.70
	168	-	-	-	-	0.000002	63.00
	169	-	-	-	-	0.0000003	85.48
Used 6	162	0.230	0.0326	0.001657	6.14	0.001231	8.33
	163	0.257	0.0393	0.000402	8.33	0.003166	10.17
	164	0.243	0.0456	0.000292	10.93	0.000051	17.00
	165	0.237	0.0525	0.000046	17.42	0.000049	25.90
	166	0.247	0.0400	0.000038	24.42	0.000011	30.38
	167	0.248	0.0411	0.000009	37.50	0.000007	46.38
	168	-	-	-	-	0.000043	68.00
	169	-	-	-	-	0.000153	86.38
